# Two-fold red excess (TREx): a simple and novel digital color index that enables non-invasive real-time monitoring of green-leaved as well as anthocyanin-rich crops

**DOI:** 10.1186/s13007-025-01339-y

**Published:** 2025-02-20

**Authors:** Avinash Agarwal, Filipe de Jesus Colwell, Viviana Andrea Correa Galvis, Tom R. Hill, Neil Boonham, Ankush Prashar

**Affiliations:** 1 School of Natural and Environmental Sciences, Newcastle University, Newcastle upon Tyne, UK; 2 Crop Science R&D Division, Infarm - Indoor Urban Farming B.V, Amsterdam, The Netherlands; 3 Human Nutrition and Exercise Research Centre, Population Health Science Institute, Faculty of Medical Sciences, Newcastle University, Newcastle upon Tyne, UK; 4 Institute for Bio- and Geosciences: Plant Sciences (IBG-2), Forschungszentrum Jülich GmbH, Jülich, Germany

**Keywords:** Image analysis, Vegetation indices, Anthocyanin, Chlorophyll, Crop monitoring, RGB

## Abstract

**Background:**

Digital color indices provide a reliable means for assessing plant status by enabling real-time estimation of chlorophyll (Chl) content, and are thus adopted widely for crop monitoring. However, as all prevalent leaf color indices used for this purpose have been developed using green-leaved plants, they do not perform reliably for anthocyanin (Anth)-rich red-leaved varieties. Hence, the present study investigates digital color indices for six types of leafy vegetables with different levels of Anth to identify congruent trends that could be implemented universally for non-invasive crop monitoring irrespective of species and leaf Anth content. For this, datasets from three digital color spaces, viz., RGB (Red, Green, Blue), HSV (Hue, Saturation, Value), and L*a*b* (Lightness, Redness-greenness, Yellowness-blueness), as well as various derived plant color indices were compared with Anth/Chl ratio and SPAD Chl meter readings of *n* = 320 leaf samples.

**Results:**

Logarithmic decline of G/R, G-minus-R, and Augmented Green-Red Index (AGRI) with increasing Anth/Chl ratio (*R*^*2*^ > 0.8) revealed that relative Anth content affected digital color profile markedly by shifting the greenness-redness balance until the Anth/Chl ratio reached a certain threshold. Further, while most digital color features and indices presented abrupt shifts between Anth-rich and green-leaved samples, the proposed color index Two-fold Red Excess (TREx) did not exhibit any deviation due to leaf Anth content and showed better correlation with SPAD readings (*R*^*2*^ = 0.855) than all other color features and vegetation indices.

**Conclusion:**

The present study provides the first in-depth assessment of variations in RGB-based digital color indices due to high leaf Anth contents, and uses the data for Anth-rich as well as green-leaved crops belonging to different species to formulate a universal digital color index TREx that can be used as a reliable alternative to handheld Chl meters for rapid high-throughput monitoring of green-leaved as well as red-leaved crops.

**Supplementary Information:**

The online version contains supplementary material available at 10.1186/s13007-025-01339-y.

## Background

Transition of emphasis from “food quantity” to “food quality” has led to a noticeable surge in interest towards expanding and improving the production of leafy vegetables in the past decade [1]. Growing focus on the nutritive value of foodstuffs has highlighted the importance of anthocyanins (Anth) as key nutritional compounds owing to their high antioxidant activity and numerous potential health benefits [2]. Consequently, there have been concerted efforts to promote large-scale production of various Anth-rich leafy vegetables belonging to diverse plant families [3]. This rising interest in cultivation of Anth-rich “red-leaved” vegetables has brought to light a new challenge for growers: monitoring the health and physiological status of such crops efficiently.

Leaf chlorophyll (Chl) content has been widely used an indicator of plant health and physiological status since decades as it is connected strongly with plant nitrogen content and directly affects photosynthesis [4–6]. Hence, non-invasive monitoring of crops via hand-held Chl meters has become very common in the past decades [7–10]. However, such devices require manual measurements from each leaf, making the process slow and labor-intensive. Further, inferences from point measurements are subjective considering localized variations in pigmentation within the leaf. As an alternative, various machine vision technologies have emerged as reliable means of high-throughput real-time monitoring of plants [11, 12]. Amongst those, RGB (Red, Green, Blue) cameras have been adopted most widely for crop monitoring considering the synergistic relation between plant health, Chl content, and leaf color [9, 13–15]. Steady improvements in RGB sensors have resulted in better resolution, reduced size, easy availability, and hassle-free application of RGB cameras, making it highly feasible for crop monitoring at a commercial scale [16–18]. Hence, RGB imaging has been especially well explored for developing digital color indices for tracking the variations in leaf color which “reflect” the physiological status of plants [19].

However, because green-leaved plants dominate conventional commercial cultivation, the existing digital color indices for crop monitoring have been developed primarily for plants with low Anth levels. Consequently, such indices focus on the total and relative abundance of Chl and carotenoids (Car) as the key indicators of the physiological status [9, 13–15, 20–23]. Conversely, digital image analysis for red-leaved (Anth-rich) plants has primarily focused on estimating leaf Anth content [24–26]. A few studies have demonstrated the feasibility of predicting Chl content in sweet potato [27] and various Anth-rich tree leaves [28, 29] using reflectance spectrophotometry. However, implementation of RGB-based color indices for monitoring green-leaved as well as Anth-rich plants in tandem remains largely unexplored.

Therefore, the current study aims to develop a universal method for monitoring both green-leaved and Anth-rich plants using RGB imaging. For this, we analyzed images from six different leafy vegetables with varying levels of Chl and Anth to visualize the impact of high Anth content on digital color features as well as established color indices. We explored the influence of changing Anth/Chl ratio on digital color parameters, to gain insights into how different pigment blends influence leaf color profiles. Subsequently, digital color attributes that remained consistent despite variations in Anth content were identified, and the information was utilized to formulate a color index that could be implemented for non-invasive real-time crop monitoring across different species and leaf Anth levels.

## Materials and methods

### Plant material

**Fig. 1:**
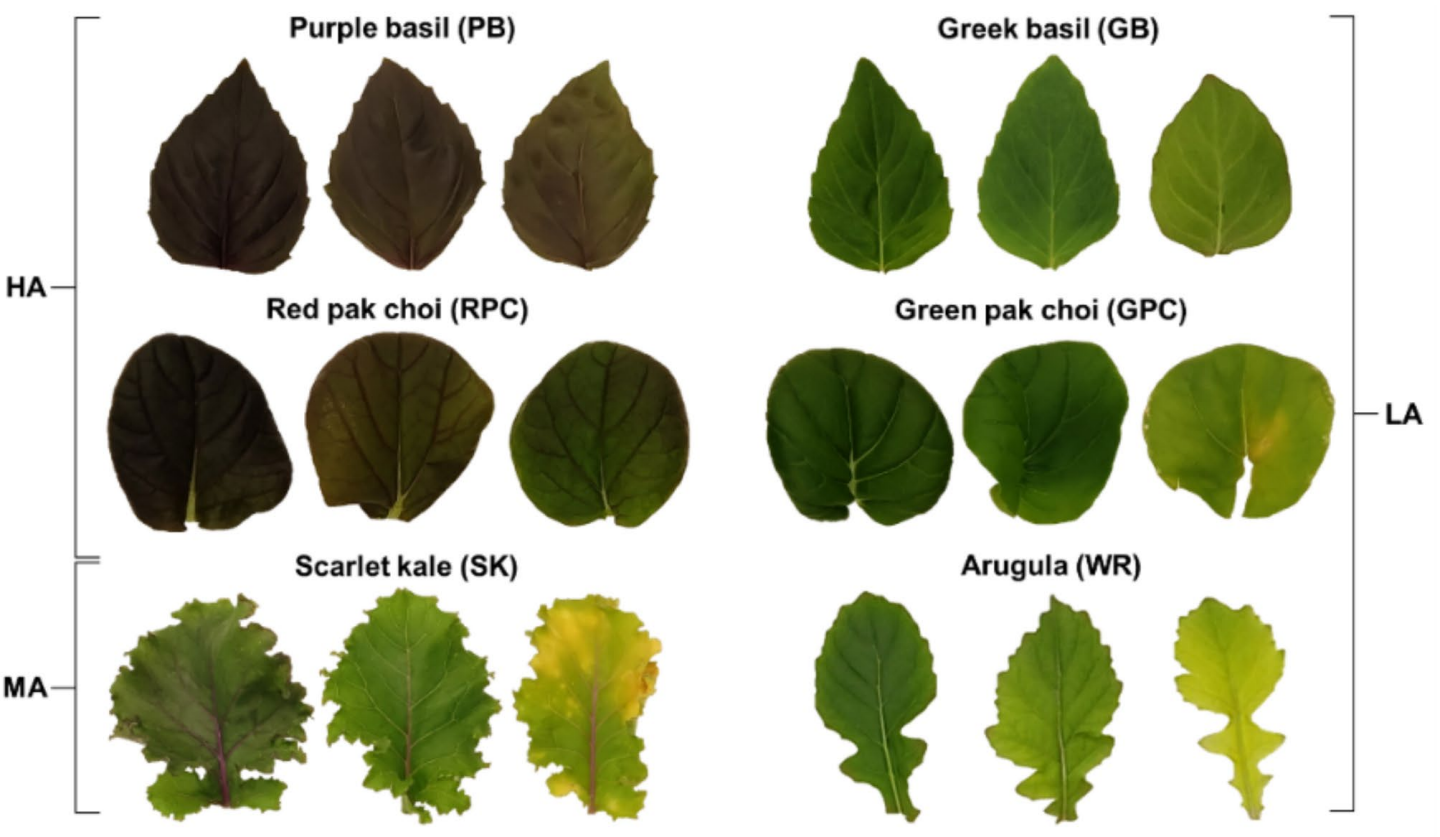
Representative leaf samples with varying levels of pigmentation from the six types of leafy vegetables selected for the present study. Purple basil and Red pak choi were grouped as high anthocyanin (HA) samples, and Scarlet kale was considered as the only member of the medium anthocyanin (MA) group. Greek basil, Green pak choi, and Arugula (cv. ‘Wasabi rocket’) were categorized as low anthocyanin (LA)

Six leafy vegetables with different levels of Anth content were selected for the present study (Fig. 1), and were classified into three groups based on leaf pigment status as follows: (1) high Anth (HA)– Purple basil (*Ocimum basilicum* L. var. *purpurascens*; PB) and Red pak choi (*Brassica rapa* L. ssp. *chinensis* cv. ‘Rubi F1’; RPC); (2) medium Anth (MA)– Scarlet kale (*Brassica oleracea* L. var. *acephala* ‘Scarlet’; SK); and (3) low Anth (LA)– Green pak choi (*Brassica rapa* L. ssp. *chinensis*; GPC), arugula (*Eruca vesicaria* ssp. *sativa* Mill. cv. ‘Wasabi Rocket’; WR), and Greek basil (*Ocimum basilicum* L. var. *minimum*; GB). Leaf color ranged between dark purple and reddish-green for HA samples, green lamina with reddish-tinge and red midrib for MA samples, and different yellow-green shades with no hint of red for LA samples (Fig. 1).

Seedlings of all plants were grown in coco-peat plugs (Van der Knapp, The Netherlands) in a nursery (Aralab-InFarm UK Ltd., London, UK), with each plug holding 5–10 seedlings. When the seedlings reached a height of ca. 5 cm, the seedling plugs were transferred to an experimental hydroponic vertical farm (InStore Farm V2, InFarm UK Ltd.) stationed at the Agriculture Building, Newcastle University, UK. Seedling plugs for each type of plant were placed in two hydroponic trays (30 × 40 cm^2^) having a 3 × 4 array of equally-spaced empty slots for seedling plugs, totaling 24 seedling plugs for each plant type. A commercial hydroponics fertilizer mix was used as the nutrient source, and irrigation was performed following the ebb-and-flow system wherein the nutrient solution was flooded into the hydroponic chamber intermittently (10 min/h) to soak the roots. A white LED array having an approximate red (400–499 nm): green (500–599 nm): blue (600–699 nm) distribution of 40:20:40 was used to provide a PPFD of 280 µmol/m^2^sec following a 16/8 h day-night cycle. Temperature and relative humidity were maintained at 25 ± 1 °C and 65 ± 5%, respectively. Sensors for temperature, humidity, flow rate, electrical conductivity, and pH within the vertical farming system were connected to a Farmboard (InFarm UK Ltd.) for real-time monitoring of the plant growth environment.

### Leaf sampling and SPAD measurement

A total of *n* = 320 leaf samples were collected from the six types of plants (PB, *n* = 60; RPC, *n* = 40; SK, *n* = 100; GPC, *n* = 40; WR, *n* = 40; GB, *n* = 40) between 15 and 20 days of growth within the experimental setup. Samples with variations in pigmentation due to inherent physiological changes were selected to get a wide range of leaf color profiles, whereas very young (< 10 days old) as well as fully-senesced leaves were specifically avoided. Leaves were labeled prior to excision, and Chl content was measured non-invasively by taking three readings from each leaf via a SPAD-502 Plus meter (Konica-Minolta, Inc., Tokyo, Japan) avoiding the midrib and prominent veins [30]. Notably, SPAD measurements have been considered the “gold standard” for non-invasive assessment of plant physiological status and estimation of leaf Chl content following numerous reports over more than two decades [10], and hence, have been used likewise in the present study. Subsequently, leaves were excised at the base for image acquisition (described in the next section), followed by destructive measurement of pigment contents (described later).

### Image acquisition

**Fig. 2:**
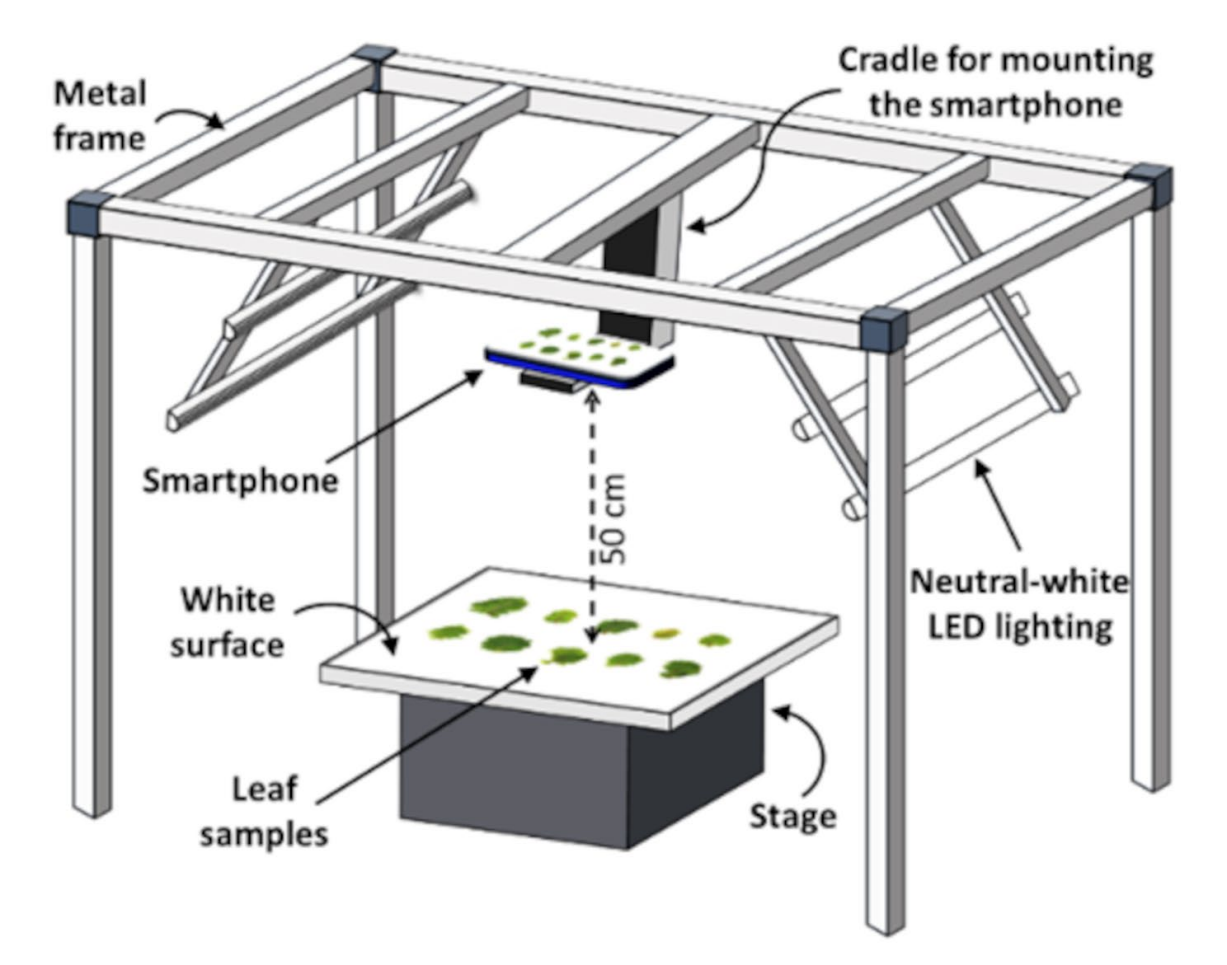
Schematic representation of the customized setup for leaf image acquisition. A metal frame was used for mounting a smartphone camera and LED lights. A matte white board was used as the background for leaf imaging while maintaining a fixed distance of 50 cm from the camera. Camera parameters (focus, exposure, and ISO) were set by focusing on the empty stage to maintain uniformity of color tone across images. Images were captured using the voice-activated mode to operate the camera remotely, avoiding camera movement and shadows

Leaf samples were transferred to a customized imaging setup for digital image acquisition (Fig. 2) immediately following excision. The setup comprised of a metal frame for mounting a smartphone camera and LED-luminaires for lighting, along with a horizontal platform (stage) with a matte white surface for placing the leaf samples. Images were acquired using a Redmi Note 7 Pro smartphone (Xiaomi Corp., Beijing, China) equipped with a Sony IMX 586 RGB sensor (size 1/2.0”, Quad-Bayer array) within a dual rear-camera system (primary lens: resolution 48 megapixels, aperture *f*/1.8, wide angle, pixel size 1.6 µm, phase detection autofocus; secondary lens: resolution 5 megapixels, aperture *f*/2.4, depth perception). The Open Camera android application (ver. 1.52, developer: Mark Harman, source: Google Play Store) was used for capturing images (8000 × 6000 pixels, sRGB color space, JPEG format). The smartphone was placed within a compact cradle suspended from the metal frame to prevent camera movement or change in camera angle. Camera-to-stage distance of 50 cm was maintained along with constant imaging parameters (exposure time 1/100 sec, ISO-200). Camera focus was fixed on the stage before placing the leaf samples, and automatic adjustments (autofocus and exposure compensation) were disabled to ensure uniformity across images. Camera flash was disabled during the process to avoid glares. Instead, lighting was provided by four neutral-white (4000 K) LED tube-lights (Model No. 0051048, Feilo Sylvania International Group Kft., Budapest, Hungary; www.sylvania-lighting.com). Fixed lighting prevented undesirable fluctuations in brightness and color temperature between images, and nullified image pre-processing requirements. As enough lighting was provided, a relatively low ISO was adequate for ensuring sharp images while minimizing noise. Images were captured remotely using the voice-activated mode within the smartphone application to avoid shadows, delays, or any disturbances that could be caused by manual handling.

### Spectrophotometric estimation of pigment contents

Leaf sections were collected for spectrophotometric estimation of total Chl, Car, and Anth contents immediately following image acquisition. Briefly, two 2 cm^2^ sections were excised from each leaf, weighed individually, sealed into separate 1.5 ml centrifuge tubes, and transferred to -20 °C for storage. Vials of all the samples were subsequently put in a liquid nitrogen bath, followed by tissue pulverization using stainless-steel beads within a tissue homogenizer (Geno/Grinder 2010, SPEX SamplePrep, Cole-Parmer, Illinois, USA). One batch of samples (*n* = 320) was used for estimating Chl and Car contents, whereas the other batch (*n* = 320) was used for estimating Anth content.

Chl and Car contents were estimated following acetone extraction as described by Lichtenthaler [31]. Briefly, 1 ml of ice-cold 80% (v/v) acetone was added to each vial, followed by centrifugation at 10,000 *g* at 4 °C for 15 min. The supernatant was collected, and the pellet was re-extracted using 1 ml of the solvent. Both supernatants were pooled, and absorbance was recorded spectrophotometrically at 470 nm (A_470_), 647 nm (A_647_), and 663 nm (A_663_) for calculating the total Chl and Car contents per unit leaf fresh weight (FW) for 2 ml (Vol) of extract as follows:

1 $$\:Chl\:\left( {mg/gFW} \right) = \frac{{\left( {18.71{A_{647}} + 7.15{A_{663}}} \right) \times \:Vol}}{{1000 \times \:FW}}\:$$

2 $$\:Car\:\left( {mg/gFW} \right) = \frac{{(1000{A_{470}} - 1822.85{A_{647}} + 411.31{A_{663}}) \times \:Vol}}{{198 \times \:1000 \times \:FW}}\:$$

Anth content was estimated following the method of Mancinelli et al. [32]. A similar extraction procedure as above was followed using chilled acidified (1% w/v HCl) methanol as the solvent. Absorbance was recorded at 530 nm (A_530_) and 657 nm (A_657_) for calculating the total Anth content using the expression A_530_– (0.25×A_657_). Here A_530_ corresponds to the peak absorbance of Anth, and A_657_ was used for pheophytin correction. A standard curve was prepared using cyanidin-3-*O*-glucoside (Merck KGaA, Darmstadt, Germany) for calculating relative Anth content per unit leaf biomass (mg/g FW).

### Color feature extraction

A customized image processing pipeline was designed to extract the color feature values of whole leaves using the *numpy* and *cv2* libraries in Python program (www.python.org). Within the pipeline, each RGB image was used to directly extract the R, G, B features using *cv2* library commands. Additionally, Hue, Saturation, and Value (HSV) as well as Lightness, Redness-greenness, and Yellowness-blueness (L*a*b*) color features were also extracted from the image. For each color feature channel, values for all pixels within the leaf boundary (minimum 5000 pixels) were averaged to obtain the mean color feature value for the entire leaf. Normalized RGB features and various previously-reported digital color indices (Table 1) were calculated using these color feature values. In addition, two new redness-based color contrast indices, viz., Simple Red Excess (SREx) and Two-fold Red Excess (TREx) were also tested (Table 1).

**Table 1:** Vegetation indices and normalized RGB features derived from digital images

Color features and indices	Equation	Reference
Normalized Red	*r* = R/(R + G + B)	[13]
Normalized Green	*g* = G/(R + G + B)	
Normalized Blue	*b* = B/(R + G + B)	
Normalized Green-Blue Difference Index	NGBDI = (G– B)/(G + B)	
Woebbecke’s Index	WI = (*g*– *b*)/(|*r*– *g*|)	[33]
Normalized Difference Pigment Index	NDPI = (R– B)/(R + B)	[34]
Normalized Difference Index	NDI = (*r*– *g*)/(*r* + *g* + 0.01)	[35]
Green-Red ratio	G/R	[36]
Green-Blue ratio	G/B	[15]
Intensity	(R + G + B)/3	
Red-Blue ratio	R/B	[37]
Green Leaf Index	GLI = (2*G– R– B)/(2*G + R + B)	[38]
Visible Atmospherically Resistance Index	VARI = (G– R)/(G + R– B)	[39]
Normalized Green-Red Difference Index	NGRDI = (G– R)/(G + R)	
Dark Green Color Index	DGCI = [1 + (H/60)– S– V]/3	[40]
Green-minus-Red	GMR = G– R	[41]
Red-plus-Green-minus-Blue	R + G– B	[15]
Excess Green	ExG = 2*G– R– B	[42]
Excess Blue	ExR = 1.4*b– *g*	[43]
Excess Red	ExB = 1.4*r– *g*	
Augmented Green-Red Index	AGRI = GMR × G/R	[44]
Red-Green difference	R– G	[45]
Red-Blue difference	R– B	
Simple Red Excess	SREx = R– G– B	-
Two-fold Red Excess	TREx = 2*R– G– B	-

B, Blue; G, Green; H, Hue; R, Red; S, Saturation; V, Value

### Data visualization and comparison

Averaged SPAD values (*n* = 3 per leaf) were plotted against the spectrophotometrically evaluated Chl content by collating the data for HA, MA, and LA samples to visualize relative trends. Similarly, Car and Anth contents were also plotted against Chl content to compare relative trends across the different sample categories. Anth/Chl ratio was compared with individual color features as well as with G/R, GMR, and AGRI to understand the impact of different pigment blends on individual color features and the greenness-redness balance. Subsequently, all remaining vegetation indices as well as RGB, HSV, L*a*b*, and *rgb* values were individually plotted against SPAD values to visualize the trends for HA, MA, and LA samples.

### Statistical analysis

Homogeneity of variance for Chl, Anth, and Car contents across the different plant species and varieties was assessed using the Bartlett test. Subsequently, Kruskal-Wallis non-parametric test and Dunn’s post hoc test were performed to assess the significance of difference amongst the different leafy vegetables for the content of each type of pigment. Curve-fitting via linear, quadratic, exponential, logarithmic, and power functions was performed in Microsoft Excel 365 (Microsoft Corp., USA) for obtaining regression trends for all scatter plots (*n* = 320). Equations along with the respective coefficient of determination (*R*^*2*^) for best-fit trendlines were selected to represent the relation mathematically. Point of inflection (elbow) for the best-fit curve of color indices in relation to Anth/Chl ratio was detected in R-software (ver. 4.0.3; www.rproject.org) within the R-Studio environment (ver. 1.3.1093; www.rstudio.com) using the *inflection* package following the Extremum Distance Estimator method.

## Results

### Pigment contents and SPAD readings

**Fig. 3:**
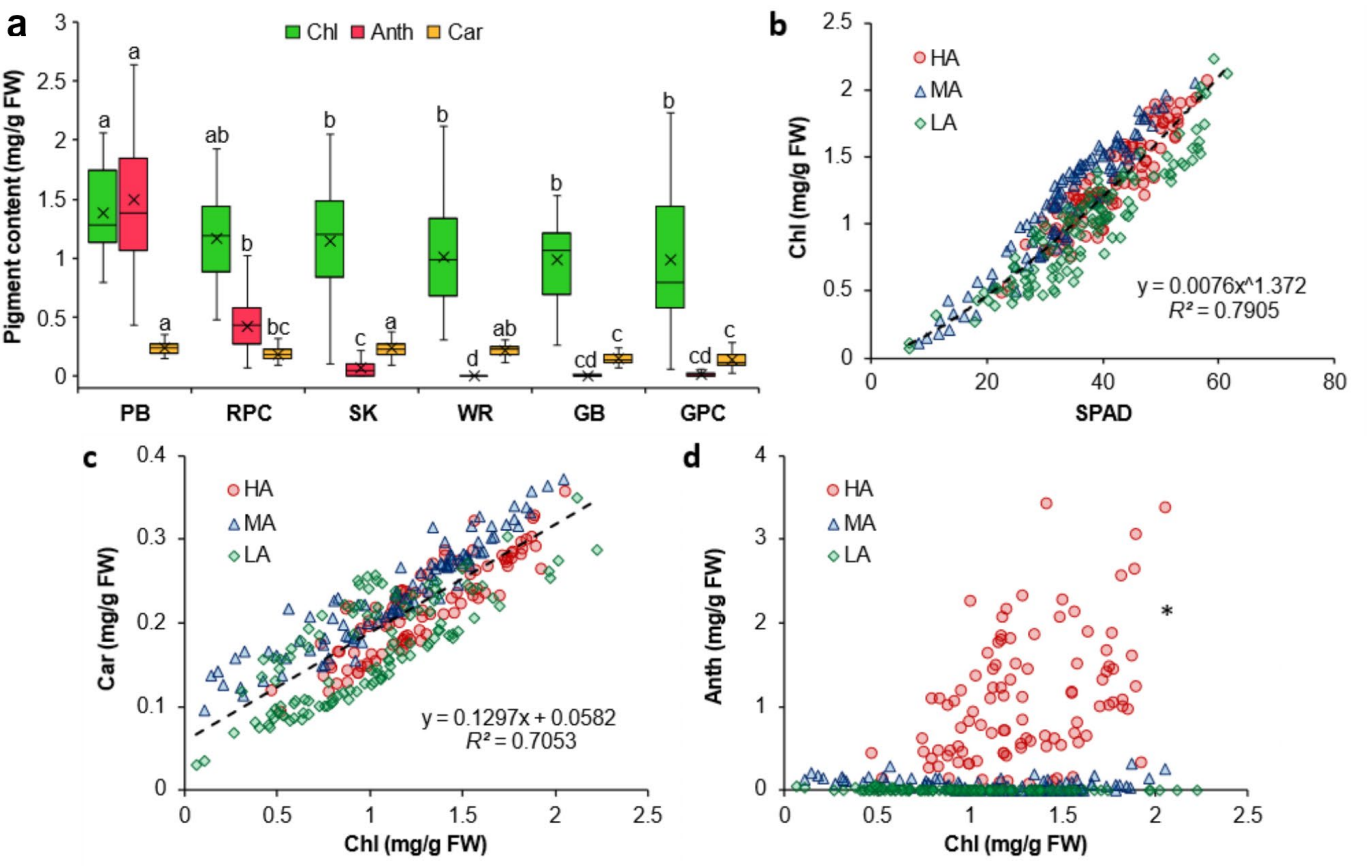
Chlorophyll (Chl), anthocyanin (Anth), and carotenoid (Car) contents of Purple basil (PB; *n* = 60), Red pak choi (RPC; *n* = 40), Scarlet kale (SK; *n* = 100), Arugula cv. ‘Wasabi rocket’ (WR; *n* = 40), Greek basil (GB; *n* = 40), and Green pak choi (GPC; *n* = 40) **(a)**, as well as the relation of Chl content with SPAD values **(b)**, Car content **(c)**, and Anth content **(d)** for plants with high (HA), medium (MA), and low (LA) levels of Anth. Box-and-Whisker plots show the mean (×), median (horizontal line), interquartile range (box), and whiskers representing 5 and 95% percentiles (a). Significant differences in mean values for each type of pigment **(a)** are indicated by different alphabets as per Dunn’s post hoc test (*p* < 0.05). Equations **(b, c)** describing the best fit curves for all data combined (*n* = 320) have been presented along with the coefficients of determination (*R*^*2*^). *Fitted curve for Chl vs. Anth **(d)** has not been presented owing to very poor correlation (*R*^*2*^ < 0.1; *n* = 320). FW, fresh weight

Chl as well as Car contents were comparable for all types of leafy vegetables, whereas Anth content varied significantly as expected (Fig. 3a). Specifically, Chl content ranged between 0.47 and 2.1 mg/g FW for the HA samples, 0.1–2.1 mg/g FW for the MA samples, and 0.06–2.3 mg/g FW for the LA samples. Similarly, Car content ranged between 0.09 and 0.36 mg/g FW for the HA samples, 0.09–0.38 mg/g FW for the MA samples, and 0.03–0.35 mg/g FW for the LA samples. In contrast, Anth content was highest in the HA plants (*p* < 0.05), with values ranging between 0.43 and 3.42 mg/g FW for PB, followed by 0.07–1.02 mg/g FW for RPC. Further, while mean Anth content for the MA group, i.e., the SK samples, was relatively closer to the lower range between 0.001 and 0.33 mg/g FW, it was lower still in the three LA plants, i.e., GB, WR, and GPC, ranging between 0.001 and 0.07 mg/g FW. SPAD values for the HA samples ranged between 22 and 59, whereas the range was 8–56 for MA samples, and 6–62 for the LA samples (Fig. 3b). A strong non-linear correlation was observed between Chl content and SPAD readings (*R*^*2*^ = 0.791; *n* = 320) upon combining the data for all samples (Fig. 3b). Similarly, the comparison between Chl and Car contents revealed a strong linear relation (*R*^*2*^ = 0.705; *n* = 320; Fig. 3c). However, no clear trend was evident between Anth and Chl contents (*R*^*2*^ < 0.1; *n* = 320), indicating a lack of correlation between the contents of these two pigments (Fig. 3d).

### Impact of Anth/Chl ratio on leaf color profile

**Fig. 4:**
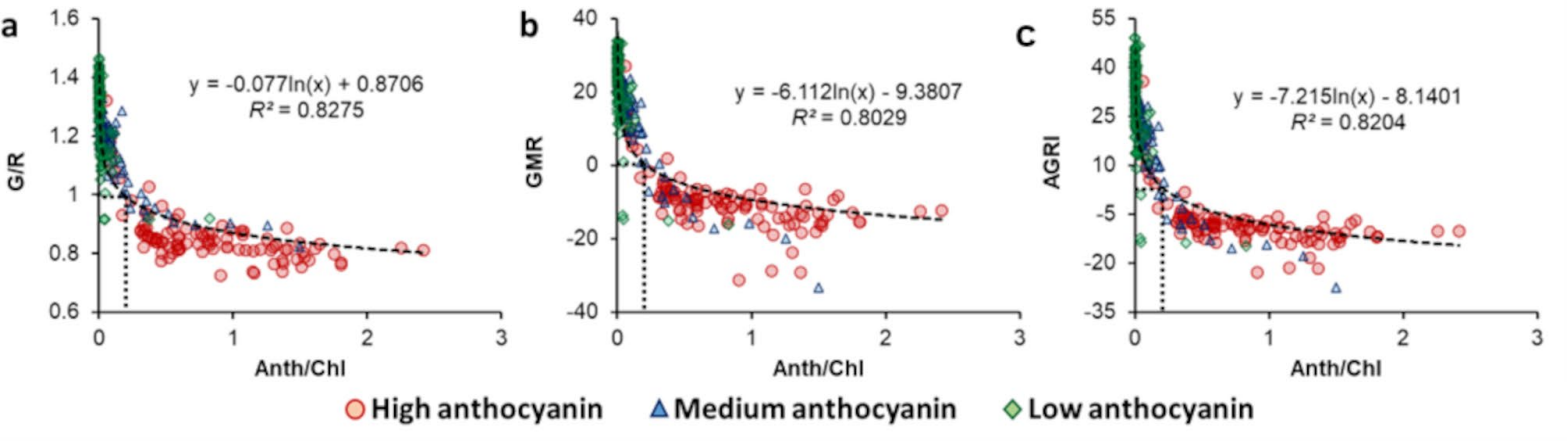
Plots for anthocyanin/chlorophyll ratio (Anth/Chl) versus Green/Red ratio (G/R; **a**), Green-minus-Red index (GMR; **b**), and Augmented Green-Red Index (AGRI; **c**) for leafy vegetables with different levels of anthocyanin (indicated with different symbols). Coefficients of determination (*R*^*2*^) and equations have been presented for the best-fit curve of the combined dataset (*n* = 320). Dotted rectangles indicate the point of inflection (elbow) in the fitted curves

Anth/Chl ratio ranged between 0.05 and 2.5 for the HA category, whereas it was generally less than 1.5 for the MA group and below 0.05 for the LA samples, with a few exceptions due to extremely low Chl in some LA leaves (Fig. 4). All three indices representing greenness-redness balance, viz., G/R, GMR, and AGRI, showed strong correlation with Anth/Chl ratio (*R*^*2*^ > 0.8; *n* = 320), wherein samples from all three groups were clustered homogenously, irrespective of leaf Anth status. Point of inflection (elbow) in the logarithmic curves was found to be at Anth/Chl ≈ 0.2 for all three indices (Fig. 4). Amongst individual color features (Supplementary Fig. S1), H showed strong correlation with Anth/Chl values (*R*^*2*^ = 0.816; *n* = 320), followed by a* (*R*^*2*^ = 0.746; *n* = 320), whereas R was affected the least by the variations in Anth/Chl ratio (*R*^*2*^ = 0.017; *n* = 320).

### Correlation of SPAD readings with color features and indices

**Fig. 5:**
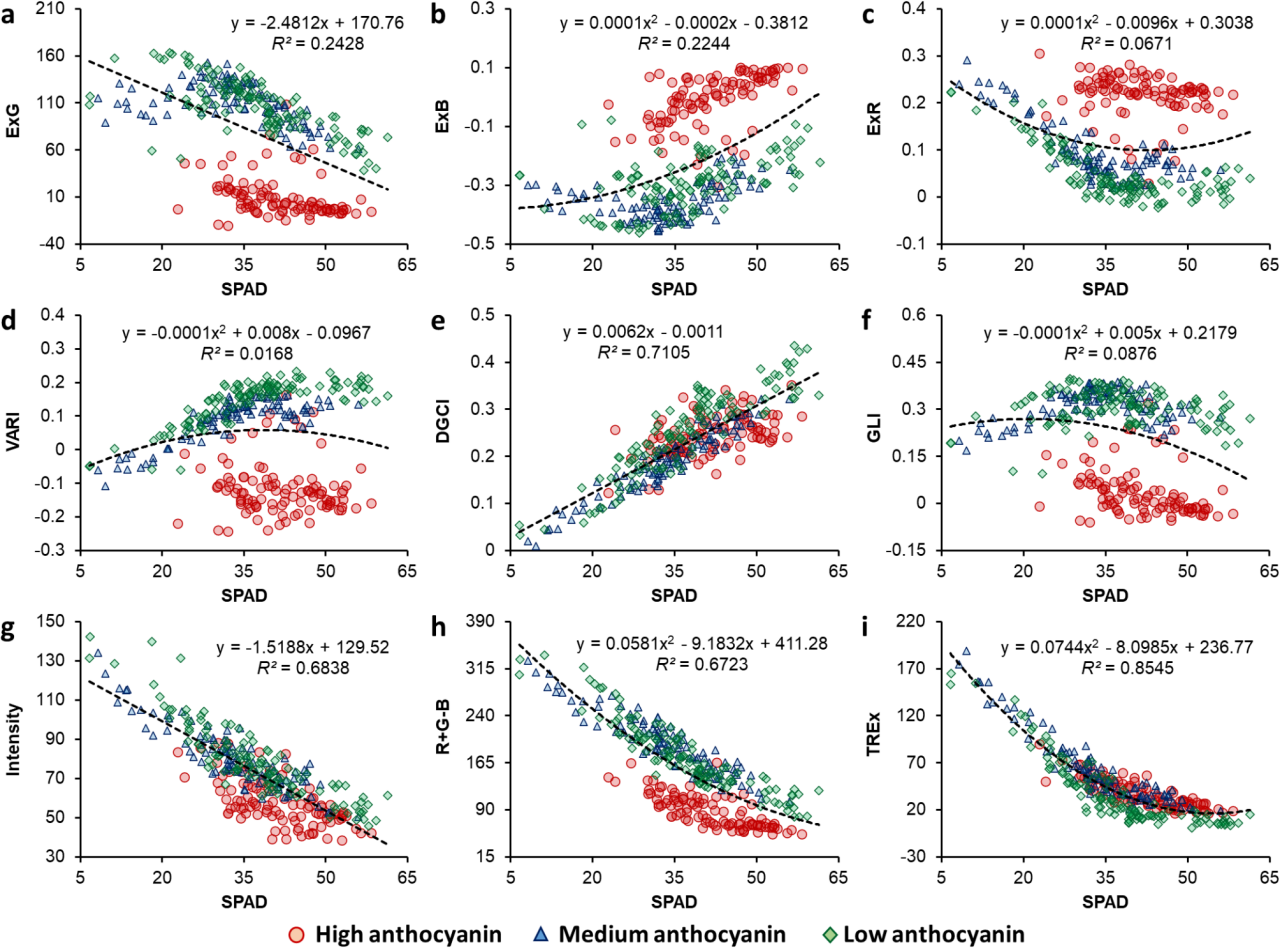
Plots for SPAD measurements versus Excess Green index (ExG; **a**), Excess Blue (ExB; **b**), Excess Red (ExR; **c**), Visible Atmospherically Resistance Index (VARI; **d**), Dark Green Color Index (DGCI; **e**), Green Leaf Index (GLI; **f**), Intensity **(g)**, Red-plus-Green-minus-Blue index (R + G-B; **h**), and Two-fold Red Excess index (TREx; **i**) for leafy vegetables with different levels of anthocyanin (indicated with different symbols). Coefficients of determination (*R*^*2*^) and equations have been presented for the best-fit curve for the combined dataset (*n* = 320)

All color indices and features revealed characteristic trends upon plotting with SPAD values (Fig. 5, Supplementary Figs. S2, S3). In general, HA samples clustered distinctly from the MA and LA samples for most of the indices as well as color features. Amongst all previously reported indices, only DGCI and Intensity exhibited good correlation with SPAD (0.68 < *R*^*2*^ < 0.72; *n* = 320) along with homogenous data distributions for the three sample categories (Fig. 5e, g). In contrast, the R-B index yielded a slightly better correlation with SPAD (*R*^*2*^ = 0.763; *n* = 320), although with minimal overlap between HA and the other two groups (Supplementary Fig. S3h). Nonetheless, correlation for all three indices was relatively weaker compared to R (*R*^*2*^ = 0.847; *n* = 320; Supplementary Fig. S2a). As an exception, only the TREx color index (*R*^*2*^ = 0.855; *n* = 320; Fig. 5i) outperformed R during SPAD correlation. Moreover, it did not show any deviation due to the presence of Anth, and a homogenous distribution of data points was observed upon collating the information for HA, MA, and LA samples.

## Discussion

### Trends in digital color profiles for varying pigment blends

In nature, leaves with adequate Chl content appear green when the concentration of green-light-absorbing pigment such as Anth is relatively low. Similarly, an Anth-rich leaf appears red only when the pigment primarily responsible for the absorption of red light, i.e., Chl, is present at low concentrations [46–48]. Accordingly, leaves with very high contents of both Chl and Anth appear dark-purplish due to strong absorption of photons across the entire visible spectrum [49, 50], as also seen in this study for the HA samples (Fig. 1). Conversely, a leaf with very low concentrations of both Chl and Anth appears “yellowish” because of high reflectance in both red and green wavebands, which indicates unmasking of Car [51]. While such variations in pigment blends impart unique colors to leaves, disentangling that information to assess the abundance of Chl remains at the focal point for all image analyses aiming at monitoring of crop health and physiological status.

Similar to previous reports on non-invasive assessment of plant physiological status [34, 51–53], the positive linear correlation between Chl and Car contents across all six types of leafy vegetables (*R*^*2*^ = 0.705; *n* = 320; Fig. 3c) indicated the strong synergy between the contents of both these pigments. Notably, such a relation also implies an increase in Car/Chl ratio with decreasing Chl content [51], which leads to leaf yellowing due to unmasking of Car pigments during leaf senescence or declining plant health [52, 54, 55]. This phenomenon alters the greenness-redness balance within the leaf color profile, and forms the basis of crop monitoring using vegetation indices typically developed using green-leaved species. However, in the present study, this trend was not perceptible due to the influence of high Anth contents (Fig. 3a), which caused a distinct shift in G values (Supplementary Fig. S2b). Hence, we focused on the Anth/Chl ratio to better understand the changes in leaf color profile in response to variations in Anth content.

In this context, selection of leaf samples from six different plant species and varieties displaying a wide range of visual color profiles (Fig. 1) enabled a comprehensive investigation of diverse combinations in digital color attributes due to variations in leaf pigment blends (Fig. 3a, c, d). Herein, the strong correlation of SPAD measurements with spectrophotometrically measured Chl contents (*R*^*2*^ = 0.791; *n* = 320; Fig. 3b) reiterated the versatility of the SPAD meter as a reliable indicator of leaf Chl status and plant health [10, 15, 35, 56], and also highlighted its uniformity across multiple plant species [7–9, 30]. Notably, there was no impact of Anth content on SPAD values (Fig. 3b), which is understandable considering that SPAD meters measure photon transmission only at 650 and 940 nm [36], i.e., within the red and infrared wavebands wherein Anth pigments are not very active.

While Chl and Anth contents showed a very poor correlation (*R*^*2*^ < 0.1; *n* = 320; Fig. 3d), comparing Anth/Chl ratio with different color features (Supplementary Fig. S1) revealed that features such as H and a*, which can indicate the balance between redness and greenness, were most strongly affected by the relative abundance of Anth and Chl (Supplementary Fig. S1d, h). Further, increasing Anth/Chl ratio caused a rapid decline in G/R, GMR, and AGRI values till G and R values converged, i.e., G/R = 1 or GMR = 0 was reached, corresponding closely with Anth/Chl = 0.2 (Fig. 4). However, R showed no deviation in response to changes in Anth/Chl ratio (Supplementary Figs. S1a, S2a). These observations indicated that although increase in leaf Anth alters the greenness-redness balance and overall leaf color profile, leaf redness remains largely unaffected. This highlights the potential of leaf redness for developing universal vegetation indices that can be implemented for monitoring the health of both green-leaved and anthocyanic crops.

### Limitations of conventional indices in broad-spectrum crop monitoring

As Chl content is a strong indicator of plant health, identification of digital color indices for crop monitoring has always focused on finding correlations with leaf Chl content estimated via leaf extracts and/or Chl-meter measurements [19, 35, 57, 58]. Indices such as DGCI [15, 22], NGRDI, VARI [35], ExG [58], and NDPI [59], as well as R and G color features [14, 15, 20, 60] have been shown to correlate well with Chl content estimates. Notably, all such studies have focused on green-leaved crops, such as soybean, potato, spinach, coffee, barley, and wheat, with hardly any reports on crops with anthocyanic leaves.

In the present study, comparison of SPAD values with these well-established color indices yielded unexpected variations in data distribution upon using the information for HA, MA, and LA samples concurrently. For example, ExG = 50, corresponding to a SPAD value of ca. 55 for green-leaved (LA) samples, was found to coincide with SPAD readings of ca. 30 for Anth-rich (HA) samples (Fig. 5a). This would imply that ExG of a healthy green-leaved plant would be the same as that of a relatively unhealthy Anth-rich plant, signifying the misleading or “red herring” effect of leaf Anth in RGB analyses. Similar trends were observed for indices such as R + G-B, NGBDI, and NDPI (Fig. 5h, Supplementary Fig. S3a, b).

Conversely, indices such as VARI, GLI, and NDI showed a bifurcation of the dataset, with LA and MA samples clustered together, but having negligible overlap with HA samples along the scale of the respective index despite similar SPAD values (Fig. 4d, f, Supplementary Fig. S3d). Another commonly-used index, viz., Woebbecke’s Excess Green (2*g*– *r*– *b*) [33], also presented a markedly similar trend as GLI with only a slight change in scale (data not shown). Such observations clearly indicate that there is a major shift in the RGB color space data which limits the scope of implementing existing RGB-based indices for monitoring green-leaved and Anth-rich crops in tandem, necessitating the development of a color index capable of this feat.

### Formulating a universal vegetation index: TREx

Since the color “green” is intuitively associated with plant health, conventional vegetation indices mostly focus on evaluating the “greenness” of leaves [33, 38, 40, 42]. Additionally, strong inverse correlation of G values with Chl content has been frequently reported as well [9, 14, 20, 58, 60]. However, in the present investigation, G values were distinctly lower in the HA (Anth-rich) samples compared to the LA and MA groups (Supplementary Fig. S2b), likely due to absorbance by Anth in the blue-green (400–599 nm) region [61]. Hence, the influence of Anth on G values makes the implementation of greenness-based color indices unreliable while monitoring the health of Anth-rich plants.

Interestingly, although leaf “redness” is commonly associated with high Anth content, it was observed that the R color feature was not affected by or correlated to Anth content at all, and displayed very good correlation with SPAD values (Supplementary Figs. S1a, S2a). This observation can be explained by the dominant absorptive activity of Chl in the red (~ 600–699 nm) waveband, where absorbance by Anth is negligible [61]. Various studies have reported a similar correlation of R with Chl content [9, 15, 20, 45, 51, 58], suggesting that reflectance within the red waveband decreases steadily with increasing Chl content. Hence, such findings suggest that assessing leaf redness by machine vision is a reliable method for monitoring plant health as compared to assessing leaf greenness.

To further explore the potential of redness-based indices for crop monitoring, three color contrast indices utilizing R, viz., R-G, R-B, and SREx (Table 1), were compared with SPAD readings (Supplementary Fig. S3g–i). While SREx and R-G presented unclear trends (*R*^*2*^ < 0.15; *n* = 320), R-B presented a data distribution that was more homogenous (*R*^*2*^ = 0.763; *n* = 320) than all other conventionally-used vegetation index in terms of HA, MA, and LA samples (Fig. 5a–h, Supplementary Fig. S3a–f). Subsequently, to test the performance of the contrast indices collectively, we combined R-B and R-G values. This fusion of both contrast indices, i.e., [R-G] + [R-B], resulted in the formulation of the TREx index, which yielded distinctly better correlation with SPAD measurements (*R*^*2*^ = 0.855; *n* = 320) while maintaining parity across all three sample categories (Fig. 5i).

While various other color contrast-based indices such as NGBDI, NGRDI, and NDPI have been found to be helpful for monitoring green-leaved crops owing to their good correlation with Chl content and SPAD measurements [19], they were found to fall short while co-analyzing green- and red-leaved samples in the present study. Moreover, although color contrast indices utilizing R have also been reported in the past, including ExG, VARI, NGRDI, and GLI, our results clearly demonstrate that TREx outperforms them all. Further, it improves the inference from R by addressing the contrast with G and B reflectance in a more balanced manner, which likely accounts for deviations in leaf color profile caused by overall elevated pigmentation due to high Anth contents more precisely. Hence, this new index has the potential to provide more accurate indications of plant physiological status, and can be utilized for real-time non-invasive high-throughput crop monitoring of green-leaved and anthocyanic crops in tandem.

### Future perspectives

As this study aimed at exploring the efficacy of vegetation indices from a new perspective by taking high Anth content into account, the experiments were performed under controlled conditions using excised leaves to obtain precise correlations between color features and SPAD measurements. The next step would be to investigate the utility of the proposed method with whole plants and in situ imaging to assess its practical feasibility for large-scale implementation. Specifically, exploring different lighting environments could enhance our understanding of how external light influences color features while using this approach. Additionally, while our study effectively utilized a limited number of HA samples with very low SPAD values, future research could benefit from using a larger number of samples from Anth-rich species with very low Chl content for a more detailed assessment of changes in RGB indices, especially at lower pigment levels. Investigating stressors to further reduce Chl content in Anth-rich species could offer valuable insights into this aspect as well. Future studies could also explore simultaneous assessment of plant health and nutritional quality via concurrently estimating Chl and Anth contents. Enhancing prediction accuracy through more advanced data processing and machine learning techniques such as deep learning could further expand the application of this approach in commercial growing systems.

## Conclusion

As the interest in cultivating Anth-rich leafy vegetables is growing steadily, improvement and optimization of high-throughput technologies for monitoring such plants has become imperative. The present study provides the first in-depth insight into the feasibility of RGB-based crop monitoring for green-leaved and anthocyanic plants simultaneously. Our findings indicated that a majority of well-established greenness-based color indices were strongly affected by high Anth content, and gave highly dissimilar outcomes for anthocyanic and green-leaved plants. However, unlike most other color features, R was not affected by leaf Anth content at all. Hence, the redness-based contrast index TREx was formulated, which was found to correlate most strongly with Chl content measured via SPAD as compared to all other color indices. Since consistent results were obtained for both green- as well as red-leaved plants belonging to four different species, our findings suggest that these colorimetric parameters have the potential to be utilized universally. Hence, by depicting the efficacy of TREx, a simple but previously unexplored digital color index, the current study provides novel insights into the utility of plant image analysis with focus on redness-based color attributes for monitoring green-leaved and Anth-rich plants alike. In-depth investigations involving additional Anth-rich crop species will further strengthen the knowledge base and broaden the applicability of this approach for assessing plant health. Expanding this research along with further validation and refinement of the method will pave the way for more widespread use in diverse agricultural settings.

## Electronic supplementary material

Below is the link to the electronic supplementary material.

Supplementary Material 1

## Data Availability

Data is provided within the manuscript or supplementary information files.

## Abbreviations

a*: Redness-greenness (L*a*b* color space)
AGRI: Augmented Green-Red Index
Anth: Anthocyanin
B: Blue channel (RGB color space)
b: Normalized Blue channel
b*: Yellowness-blueness (L*a*b* color space)
Car: Carotenoid
Chl: Chlorophyll
DGCI: Dark Green Color Index
ExG: Excess Green
G: Green channel (RGB color space)
g: Normalized Green channel
GB: Greek basil
GLI: Green Leaf Index
GMR: Green-minus-Red
GPC: Green pak choi
H: Hue (HSV color space)
HA: High anthocyanin
HSV: Hue, Saturation, Value color space
L*: Lightness (L*a*b* color space)
LA: Low anthocyanin
L*a*b*: Lightness, Redness-greenness, Yellowness-blueness color space
MA: Medium anthocyanin
NDPI: Normalized Difference Pigment Index
NExG: Normalized Excess Green
NGBDI: Normalized Green Blue Difference Index
NGRDI: Normalized Green Red Difference Index
PB: Purple basil
R: Red channel (RGB color space)
r: Normalized Red channel
RGB: Red, Green, Blue color space
RPC: Red pak choi
RMSE: Root-mean-squared error
S: Saturation (HSV color space)
SK: Scarlet kale
SPAD: SPAD-502 chlorophyll meter
SREx: Simple Red Excess
TREx: Two-fold Red Excess
V: Value (HSV color space)
VARI: Visible Atmospherically Resistance Index
WR: Arugula cv. ‘Wasabi Rocket’
