# Correction: Two-fold red excess (TREx): a simple and novel digital color index that enables non-invasive real-time monitoring of green-leaved as well as anthocyanin-rich crops

**DOI:** 10.1186/s13007-025-01370-z

**Published:** 2025-05-09

**Authors:** Avinash Agarwal, Filipe de Jesus Colwell, Viviana Andrea Correa Galvis, Tom R. Hill, Neil Boonham, Ankush Prashar

**Affiliations:** 1 School of Natural and Environmental Sciences, Newcastle University, Newcastle Upon Tyne, UK; 2 Crop Science R&D Division, Infarm - Indoor Urban Farming B.V, Amsterdam, The Netherlands; 3 Human Nutrition and Exercise Research Centre, Population Health Science Institute, Faculty of Medical Sciences, Newcastle University, Newcastle Upon Tyne, UK; 4 Institute for Bio- and Geosciences: Plant Sciences (IBG-2), Forschungszentrum Jülich GmbH, Jülich, Germany

**Correction: Plant Methods (2025) 21:24** 10.1186/s13007-025-01339-y

In this article Figs. 2, 3 and 4 appeared incorrectly and have now been corrected in the original publication. For completeness and transparency, the old incorrect versions are displayed below.

Incorrect Fig. 2
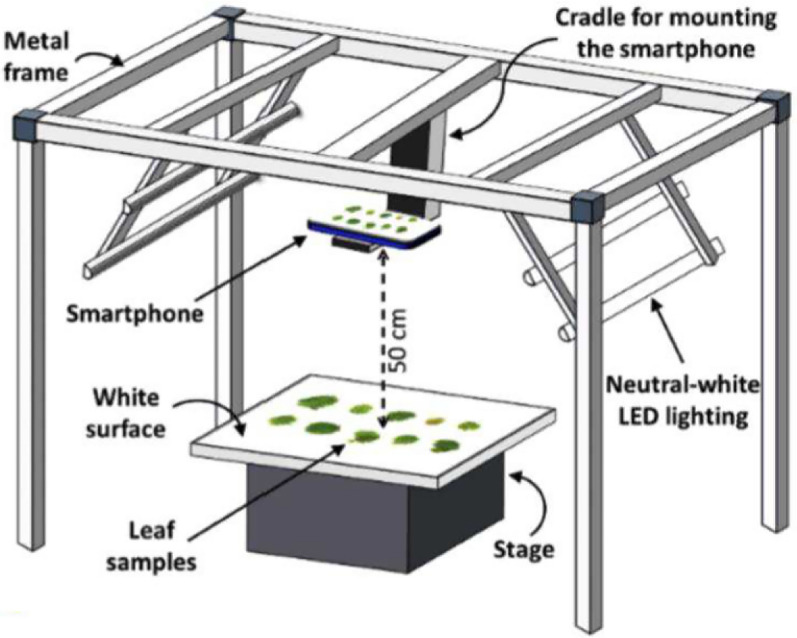


Corrected Fig. 2

**Fig. 2:**
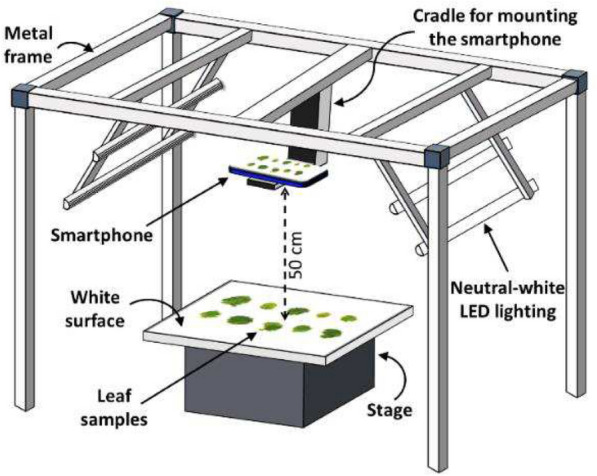
Schematic representation of the customized setup for leaf image acquisition. A metal frame was used for mounting a smartphone camera and LED lights. A matte white board was used as the background for leaf imaging while maintaining a fixed distance of 50 cm from the camera. Camera parameters (focus, exposure, and ISO) were set by focusing on the empty stage to maintain uniformity of color tone across images. Images were captured using the voice-activated mode to operate the camera remotely, avoiding camera movement and shadows

Incorrect Fig. 3
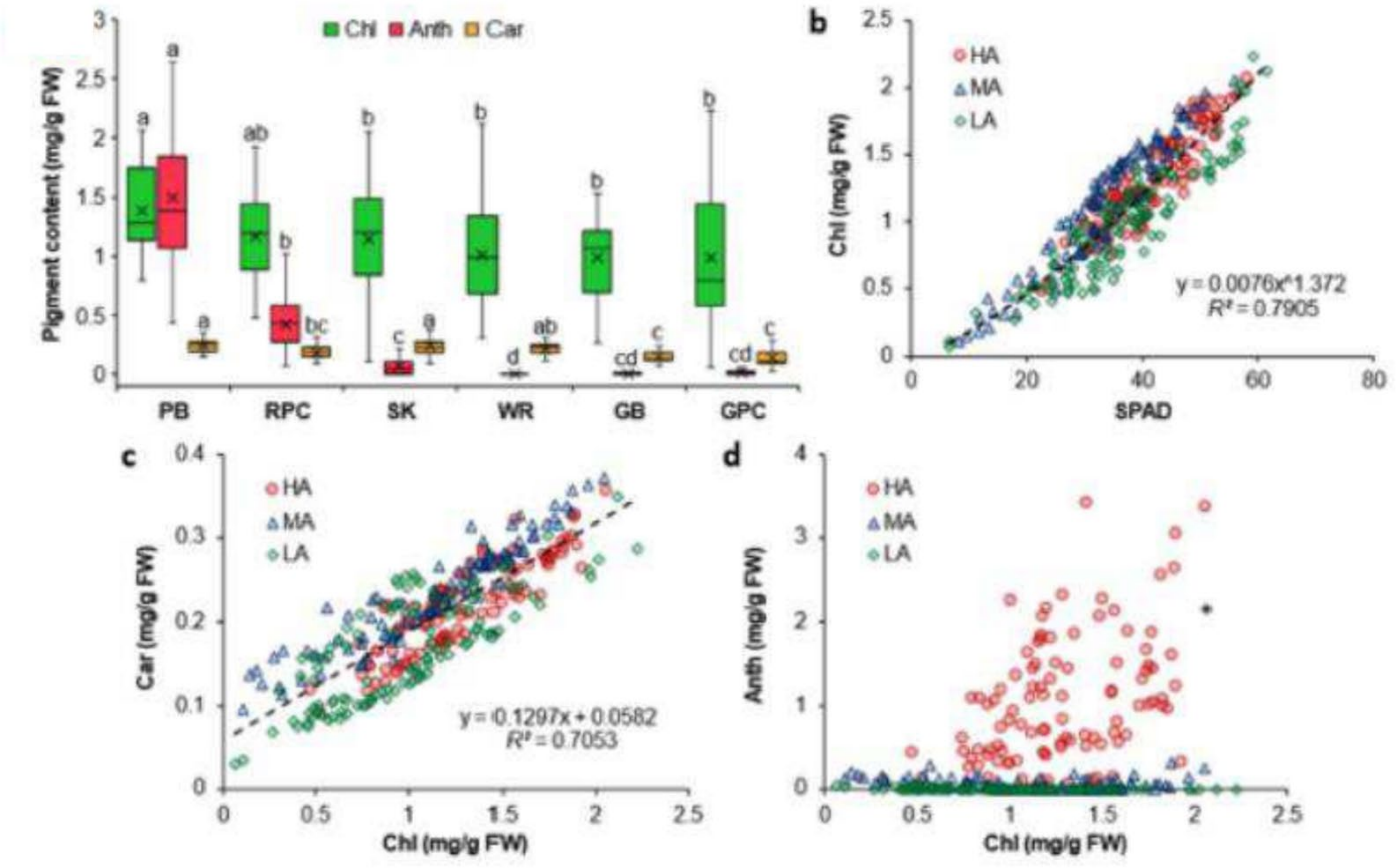


Correct Fig. 3

**Fig. 3:**
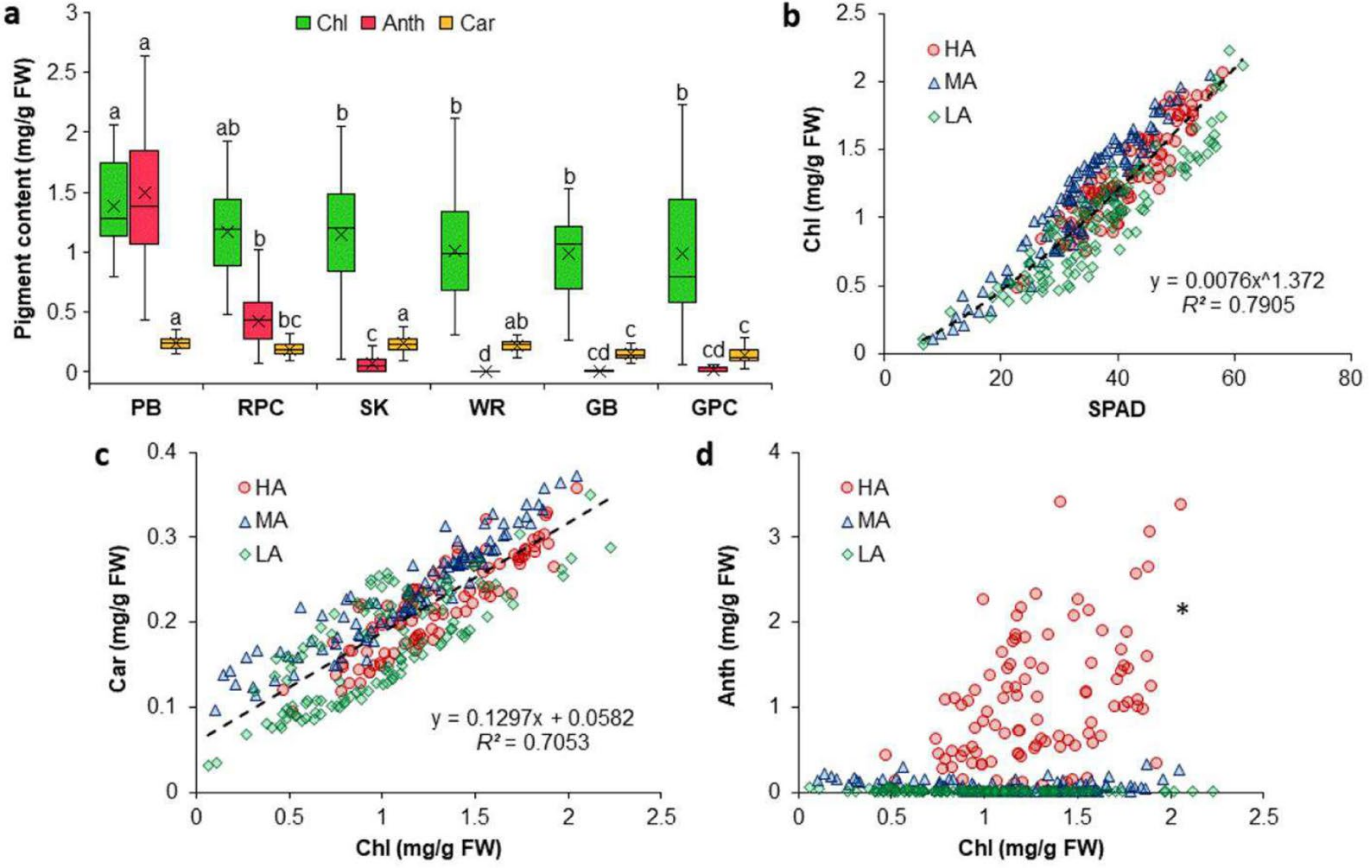
Chlorophyll (Chl), anthocyanin (Anth), and carotenoid (Car) contents of Purple basil (PB; *n* = 60), Red pak choi (RPC; *n* = 40), Scarlet kale (SK; *n* = 100), Arugula cv. ‘Wasabi rocket’ (WR; *n* = 40), Greek basil (GB; *n* = 40), and Green pak choi (GPC; *n* = 40) (**a**), as well as the relation of Chl content with SPAD values (**b**), Car content (**c**), and Anth content (**d**) for plants with high (HA), medium (MA), and low (LA) levels of Anth. Box-and-Whisker plots show the mean (×), median (horizontal line), interquartile range (box), and whiskers representing 5 and 95% percentiles (**a**). Significant differences in mean values for each type of pigment (**a**) are indicated by different alphabets as per Dunn’s post hoc test (*p* < 0.05). Equations (**b**, **c**) describing the best fit curves for all data combined (*n* = 320) have been presented along with the coefficients of determination (*R*^*2*^). *Fitted curve for Chl vs Anth (**d**) has not been presented owing to very poor correlation (*R*^*2*^ < 0.1;* n* = 320). *FW* fresh weight

Incorrect Fig. 4
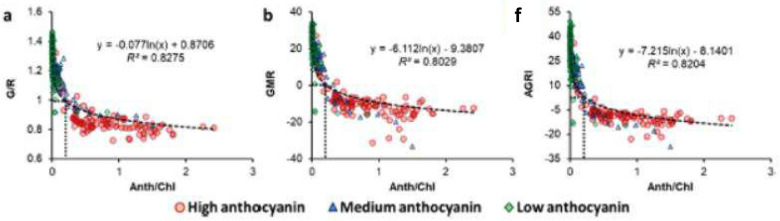


Correct Fig. 4

**Fig. 4:**
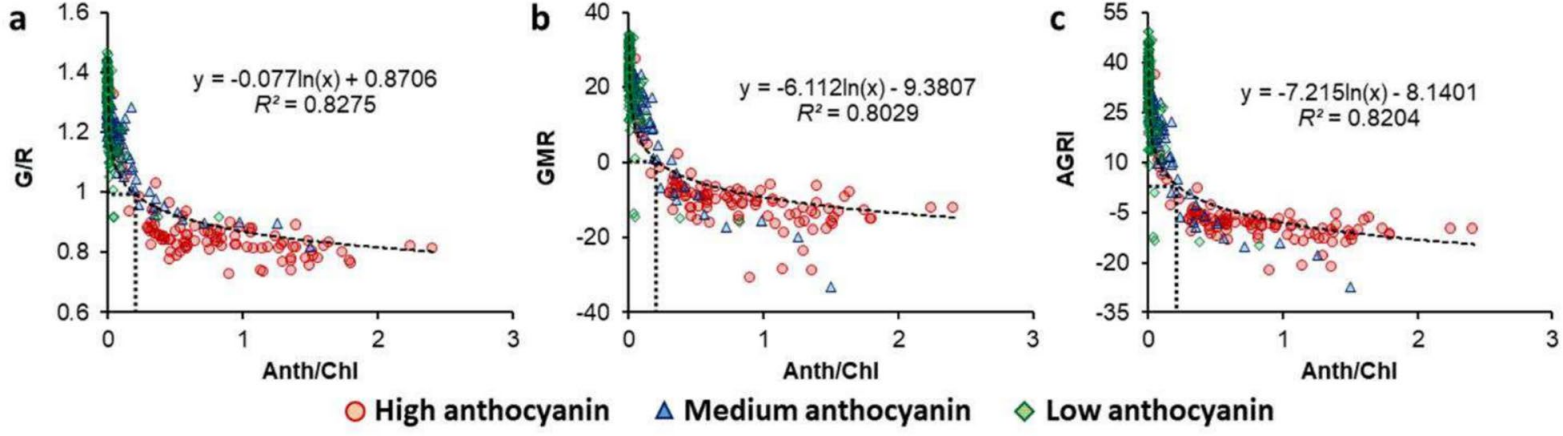
Plots for anthocyanin/chlorophyll ratio (Anth/Chl) versus Green/Red ratio (G/R; **a**), Green-minus-Red index (GMR; **b**), and Augmented Green–Red Index (AGRI; **c**) for leafy vegetables with different levels of anthocyanin (indicated with different symbols). Coefficients of determination (*R*^*2*^) and equations have been presented for the best-fit curve of the combined dataset (*n* = 320). Dotted rectangles indicate the point of inflection (elbow) in the fitted curves

The original article has been corrected.

