# Supplementary material for: Two-fold red excess (TREx): a simple and novel digital color index that enables non-invasive real-time monitoring of green-leaved as well as anthocyanin-rich crops
Source: Plant Methods. 2025 Feb 20;21:24. doi: 10.1186/s13007-025-01339-y (PMC11843946; doi:10.1186/s13007-025-01339-y)
Supplement: Supplementary file 1 — Supplementary Material 1 [file 13007_2025_1339_MOESM1_ESM.docx]

**Two-fold Red Excess (TREx): A simple and novel digital color index that enables non-invasive real-time monitoring of green-leaved as well as anthocyanin-rich crops**

Avinash Agarwal^1,4^*, Filipe de Jesus Colwell^2^, Viviana Andrea Correa Galvis^2^, Tom R. Hill^3^, Neil Boonham^1^, Ankush Prashar^1^*

*^1^School of Natural and Environmental Sciences, Newcastle University, Newcastle upon Tyne, UK*

*^2^Crop Science R&D Division, Infarm - Indoor Urban Farming B.V., Amsterdam, The Netherlands*

*^3^Human Nutrition and Exercise Research Centre, Population Health Science Institute, Faculty of Medical Sciences, Newcastle University, Newcastle upon Tyne, UK*

*^4^Institute of Bio- and Geosciences: Plant Sciences (IBG-2), Forschungszentrum Jülich GmbH, Jülich, Germany*

***SUPPLEMENTARY MATERIAL***

***Supplementary figures***


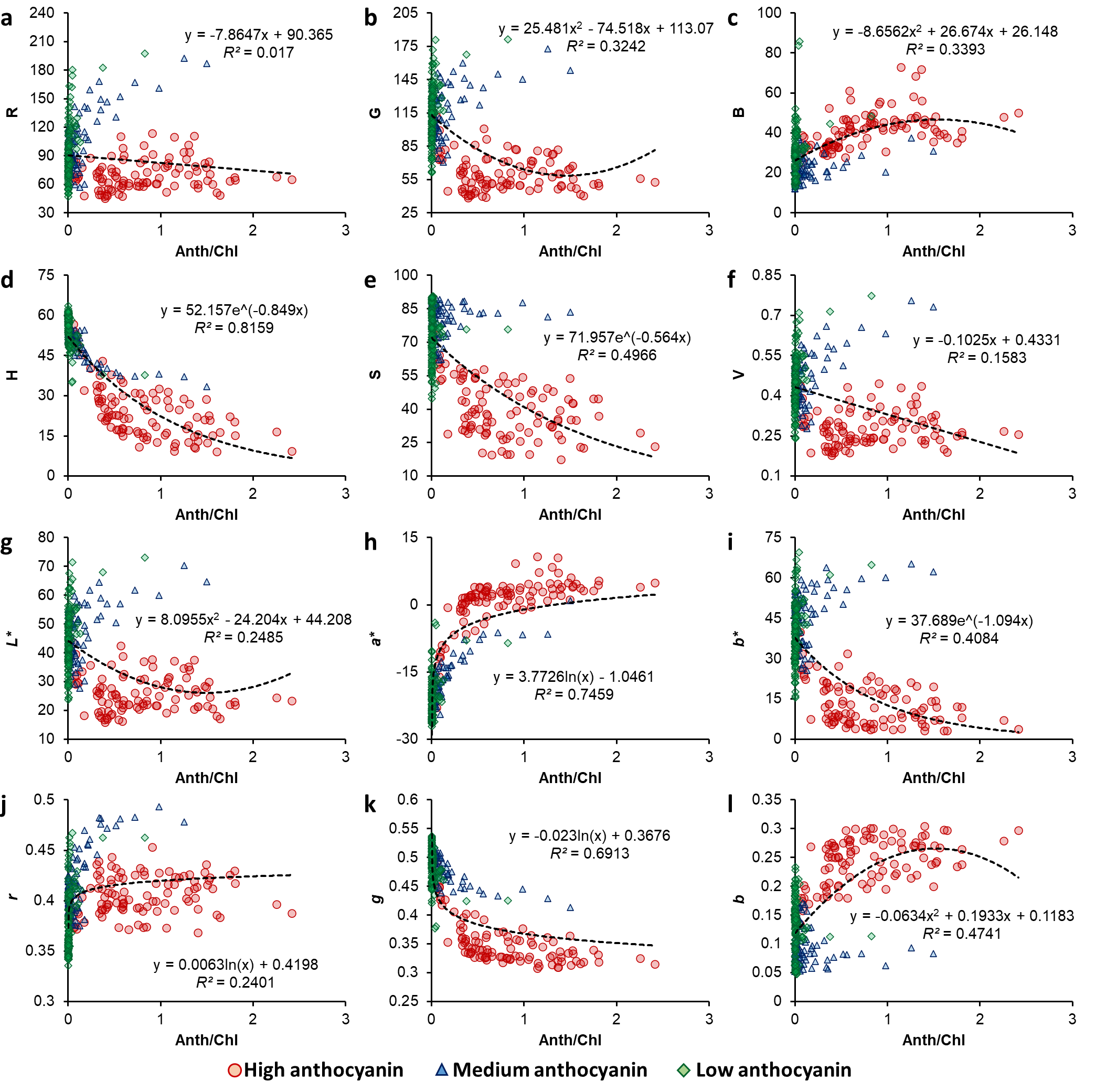


**Fig. S1** Plots of anthocyanin/chlorophyll ratio (Anth/Chl) with different digital color features, i.e., Red (R), Green (G), Blue (B) (a–c), Hue (H), Saturation (S), Value (V) (d–f), Lightness (*L**), Redness-greenness (*a**), Yellowness-blueness (*b**) (g–i), normalized Red (*r*), normalized Green (*g*), and normalized Blue (*b*) (j–l), for leaves with different levels of anthocyanin content. Coefficients of determination (*R^2^*) and equations have been presented for the best-fit curve (*n* = 320).


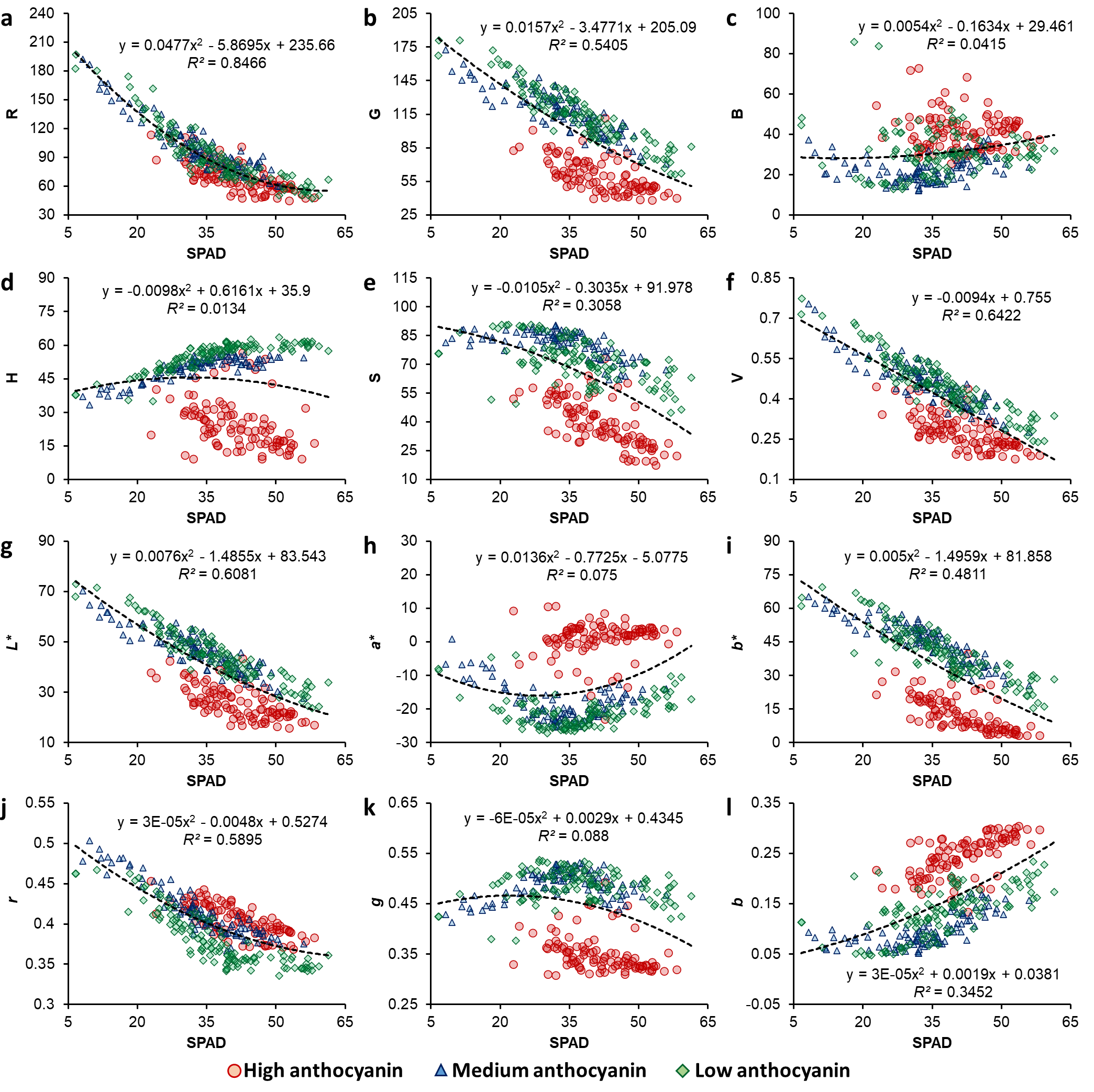


**Fig. S2** Plots of SPAD measurements with different digital color features, i.e., Red (R), Green (G), Blue (B) (a–c), Hue (H), Saturation (S), Value (V) (d–f), Lightness (*L**), Redness-greenness (*a**), Yellowness-blueness (*b**) (g–i), normalized Red (*r*), normalized Green (*g*), and normalized Blue (*b*) (j–l), for leaves with different levels of anthocyanin content. Coefficients of determination (*R^2^*) and equations have been presented for the best-fit curve (*n* = 320).


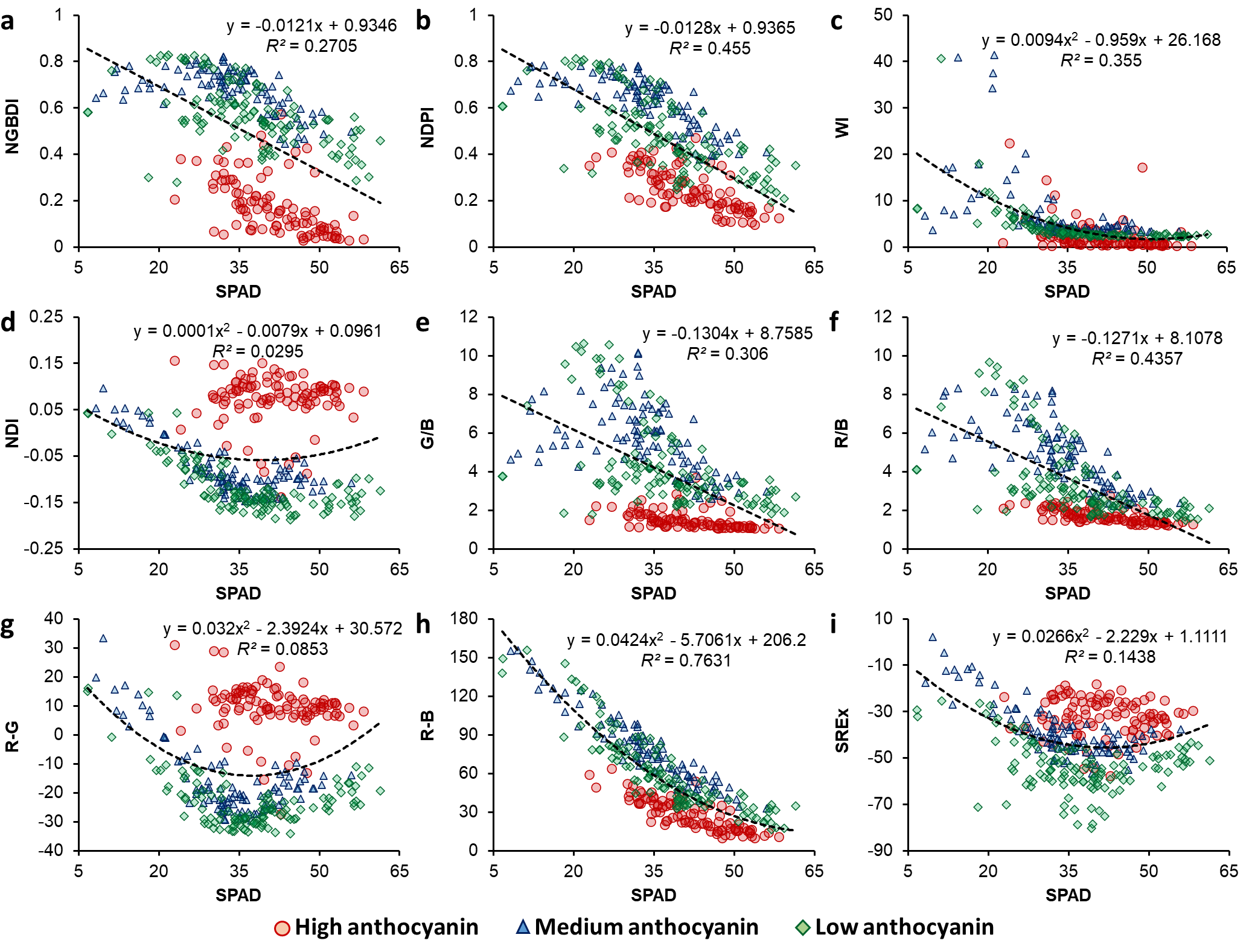


**Fig. S3** Plots for SPAD measurements versus Normalized Green-Blue Difference Index (NGBDI; a), Normalized Difference Pigment Index (NDPI; b), Woebbecke’s Index (WI; c), Normalized Difference Index (NDI; d), Green-Blue ratio (G/B; e), Red-Blue ratio (R/B; f), Red-Green difference (R-G; g), Red-Blue difference (R-B; h), and Simple Red Excess index (SREx; i) for leafy vegetables with different levels of anthocyanin (indicated with different symbols). Coefficients of determination (*R^2^*) and equations have been presented for the best-fit curve for the combined dataset (*n* = 320).
